# Supplementary figures and images for: Clinical Implication of Concurrent Amplification of MET and FGFR2 in Metastatic Gastric Cancer
Source: Biomedicines. 2023 Nov 28;11(12):3172. doi: 10.3390/biomedicines11123172 (PMC10740780; doi:10.3390/biomedicines11123172)

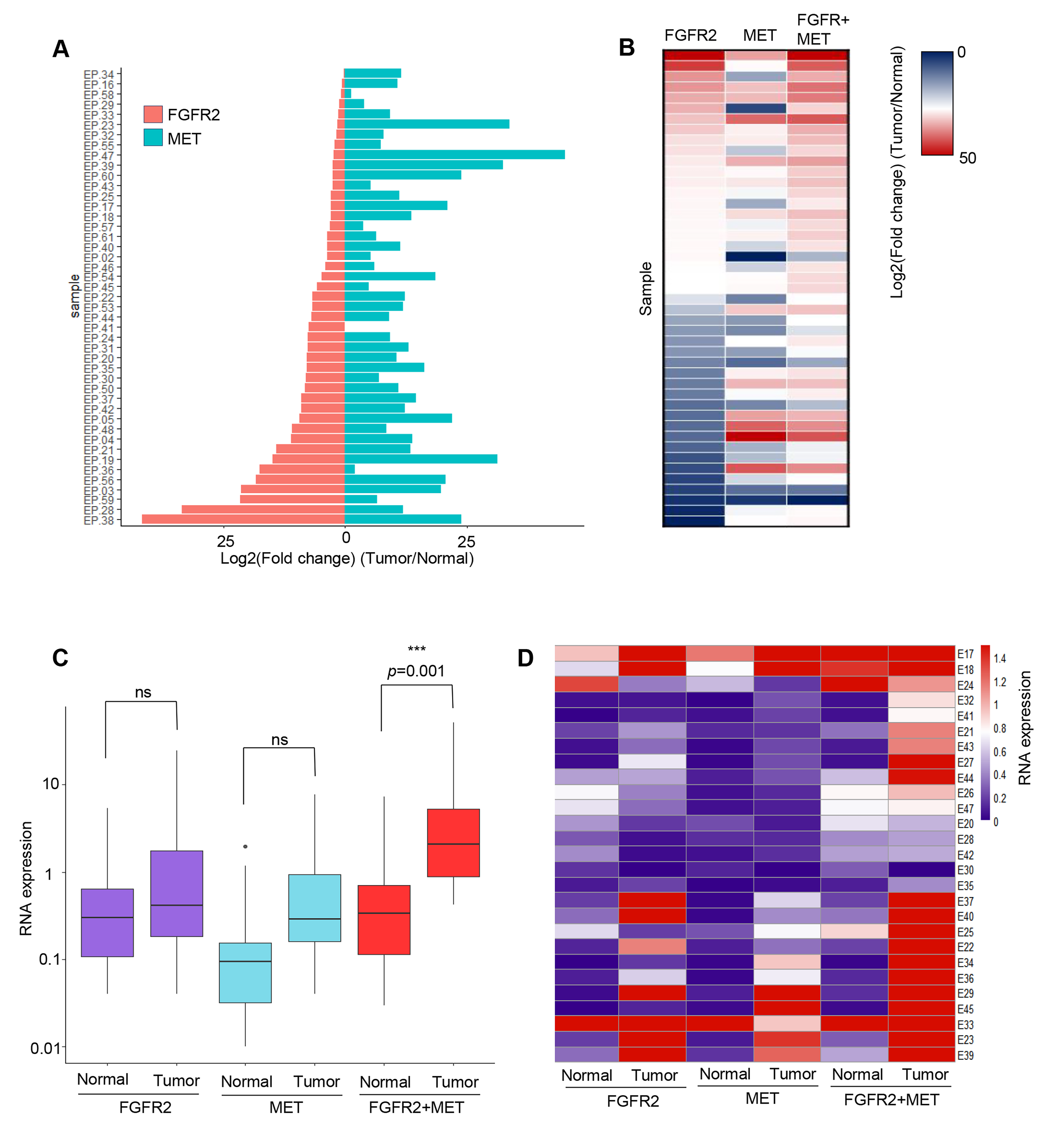

Supplement: Supplementary file 1 [file biomedicines-11-03172-s001.zip › Supple. Figure S1 (231128).tif]

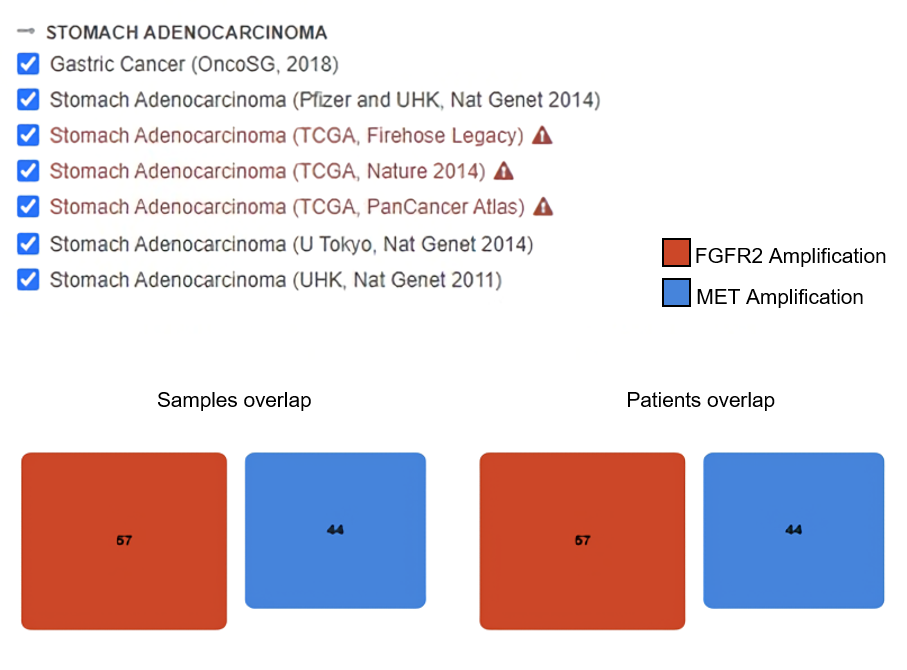

Supplement: Supplementary file 1 [file biomedicines-11-03172-s001.zip › Supple. Figure S2_high_resolution3.png]
